# Safety, tolerability, pharmacokinetics, and pharmacodynamics of single and multiple doses of aficamten in healthy Chinese participants: a randomized, double-blind, placebo-controlled, phase 1 study

**DOI:** 10.3389/fphar.2023.1227470

**Published:** 2023-08-23

**Authors:** Xue Zhao, Hongzhong Liu, Wei Tian, Ligang Fang, Mengyang Yu, Xiaofei Wu, Aijing Liu, Ruijie Wan, Li Li, Jinghui Luo, Yuqiong Li, Bo Liu, Yu He, Xiaowen Chen, Yuan Li, Donghong Xu, Hongyun Wang, Xiaohong Han

**Affiliations:** ^1^ Clinical Pharmacology Research Center, Peking Union Medical College Hospital, Beijing, China; ^2^ Internal Medicine-Cardiovascular Department, Peking Union Medical College Hospital, Beijing, China; ^3^ Ji Xing Pharmaceuticals (Shanghai) Co., Ltd., Shanghai, China; ^4^ Cytokinetics, Incorporated, South San Francisco, CA, United States

**Keywords:** aficamten, Chinese participants, safety, pharmacokinetics, pharmacodynamics

## Abstract

**Objectives:** Aficamten is a selective, small-molecule allosteric inhibitor of cardiac sarcomere being developed as a chronic oral treatment for patients with symptomatic obstructive hypertrophic cardiomyopathy. This was the first-in-Chinese study aiming to investigate the safety, tolerability, pharmacokinetics, and pharmacodynamics of aficamten in healthy adults.

**Methods:** This double-blind, randomized, placebo-controlled, phase 1 study was conducted in 28 healthy male and female Chinese participants after single ascending dose (SAD) and multi-dose (MD) administrations of aficamten. In the SAD cohort, 16 participants were randomized to receive a single oral dose of aficamten: 10 mg, 20 mg, or placebo. In the MD cohort, 12 participants were randomized to receive multiple doses of aficamten: 5 mg or placebo once daily for 14 days. Safety was monitored throughout the study with electrocardiograms, echocardiograms, clinical laboratory tests, and reporting of adverse events (AEs). Pharmacokinetic profiles of aficamten and metabolites, as well as CYP2D6 genetic impact, were evaluated.

**Results:** A total of 35 treatment-emergent AEs were reported by 14 (50%) participants with mild severity. There were no serious AEs or adverse decreases in left ventricular ejection fraction below 50% during the study. Aficamten was dose-proportional over the dose range of 5–20 mg and accumulated in the MD cohort.

**Conclusion:** Aficamten was safe and well-tolerated in the healthy Chinese adult participants. The pharmacokinetics of aficamten in the Chinese population was comparable to those previously found in Western participants. These phase 1 data support the progression of aficamten into future clinical studies in Chinese patients.

**Clinical Trial registration:** https://clinicaltrials.gov, identifier: NCT04783766.

## 1 Introduction

Hypertrophic cardiomyopathy (HCM) is one of the most common inherited cardiovascular disorders, affecting approximately 1/500 worldwide (Maron et al., 1995) and 80/100,000 in China (Zou et al., 2004). It is estimated that there are more than 1 million adult HCM patients in China. HCM is a heterogeneous disorder with marked diversity in clinical expression, natural history, and prognosis. Approximately 70% of patients with phenotypic HCM will demonstrate an element of left ventricular outflow tract (LVOT) obstruction (Maron et al., 2006). The mechanisms for developing obstruction are defined and involve a complex interplay between alterations in ventricular flow between asymmetric septal hypertrophy and the mitral valve leaflets. Currently, the main approaches to the treatment of obstructive HCM (oHCM) are medical management and septal reduction therapy. Guideline-directed pharmacologic options include beta-blockers, calcium-channel blockers, and disopyramide; however, the magnitude of benefit to patients is variable, and more invasive treatments (surgical myomectomy or septal ablation) are often necessary to treat individuals with substantial symptoms or functional deficits (Elliott et al., 2014; Ponikowski et al., 2016; Guideline writing group for the diagnosis and treatment of adult hypertrophic cardiomyopathy in Chinese cardiology society of Chinese medical association EboCjocd, 2017; Heart failure committee of Chinese medical doctor association EbotCjohfac, 2017; Ommen et al., 2020). Contemporary management strategies for oHCM have resulted in the majority of patients achieving normal or near-normal longevity and improved morbidity; however, there has been little progress with the development of novel pharmacotherapies.

HCM results from pathogenic genetic mutations, often affecting the genes encoding the proteins of the cardiac sarcomere, such as myosin (Maron, 2018; Sharpe et al., 2023). Mechanistically, mutations in HCM appear to increase the net power generation in the sarcomere *in vitro* (Chuan et al., 2012; Sommese et al., 2013; Spudich et al., 2016; Toepfer et al., 2019). The findings in these studies are consistent with the underlying myocardial pathophysiology of the left ventricle (LV) in patients with HCM being hypercontractile with diminished compliance (Wilson et al., 1967). These investigations have enhanced understanding of the molecular pathogenesis of HCM and have stimulated efforts designed to identify cardiac myosin modulators that can target the underlying mechanism of hypercontractility in obstructive HCM (Masri and Olivotto, 2022).

Aficamten (formerly CK-3773274), a next-in-class selective and small molecule allosteric inhibitor of cardiac myosin, is being developed as a chronic oral treatment for patients with HCM ^17^. Aficamten is designed to reduce the hypercontractility that underlies the pathophysiology of HCM in the cardiac sarcomere. The intended pharmacologic effect is the reduction in force produced by the cardiac sarcomere resulting in the reduction of LVOT obstruction and improved diastolic function in patients with oHCM. The reduction in cardiac contractile force is expected to alleviate left ventricular outflow tract (LVOT) obstruction in oHCM patients by counteracting the excessive thickening of the left ventricular wall of the outflow tract.

Based on the preclinical profile (Chuang et al., 2021), aficamten was further investigated in a series of human clinical studies, including a first-in-human (FIH) phase 1 study in healthy adult Western participants (CY 6011; NCT03767855) (Malik et al., 2022) and a phase 2 study in patients with HCM (CY 6021; NCT04219826) (Maron et al., 2023; ClinicalTrials.gov: NCT04219826, 2022). A multi-center phase 3 worldwide study, including China, evaluating the efficacy and safety of aficamten in patients with oHCM is ongoing (CY 6031 [SEQUOIA-HCM]; NCT05186818) (ClinicalTrials.gov: NCT05186818, 2022). Results of the FIH phase 1 study (Robertson et al., 2019; Malik et al., 2022) demonstrated aficamten to be safe and well-tolerated in healthy Western populations. The criteria for stopping dose escalation were reached after a single dose of 75 mg and after 14 days of a daily dose of 10 mg. Pharmacokinetics (PK) exposures were generally dose linear, and the steady-state appeared evident after 14 days of daily dosing. *In vitro*, cytochrome P450 (CYP) phenotyping implicated that CYP2D6, as the primary isoform involved in aficamten metabolism, contributed to the formation of major metabolites CK-3834282 and CK-3834283. However, data from clinical study CY 6011 suggested that poor metabolizers of CYP2D6 did not affect the pharmacokinetics of aficamten.

Based on previous preclinical and clinical study results, aficamten has the potential to become the next-in-class cardiac myosin inhibitor. However, the lack of clinical data in Asian populations, especially Chinese, limits its application in Asia since different populations may exhibit different drug sensitivities. It is recognized that both intrinsic and extrinsic factors may impact the PK, safety, and dose-response in different ethnic populations. Therefore, this randomized, double-blind, placebo-controlled, phase 1 study was designed to evaluate the safety and tolerability of single and multiple doses of aficamten administered orally to healthy Chinese adults to evaluate the pharmacokinetic profile of aficamten and metabolites, as well as to describe the pharmacokinetics (PK)–Pharmacodynamics (PD) relationship of aficamten and cardiac function. This was the first step for further investigation of the characteristics and applicability of aficamten in Chinese patients with obstructive HCM.

## 2 Materials and methods

Before conducting this study, the Ethics Committee at the clinical site (Peking Union Medical College Hospital, Beijing, China) granted approval for the final protocol and amendments. The Human Genetic Resource Administration of China approved the quantity of the blood samples collected in this study. All participants provided written informed consent prior to enrollment. This study was conducted in compliance with the local laws and regulations and in accordance with the Declaration of Helsinki, the International Council for Harmonization E6 (R2) Good Clinical Practice guidelines, and the sponsor’s policy on bioethics. The study was registered on ClinicalTrials.gov (NCT04783766).

### 2.1 Study design

**FIGURE 1:**
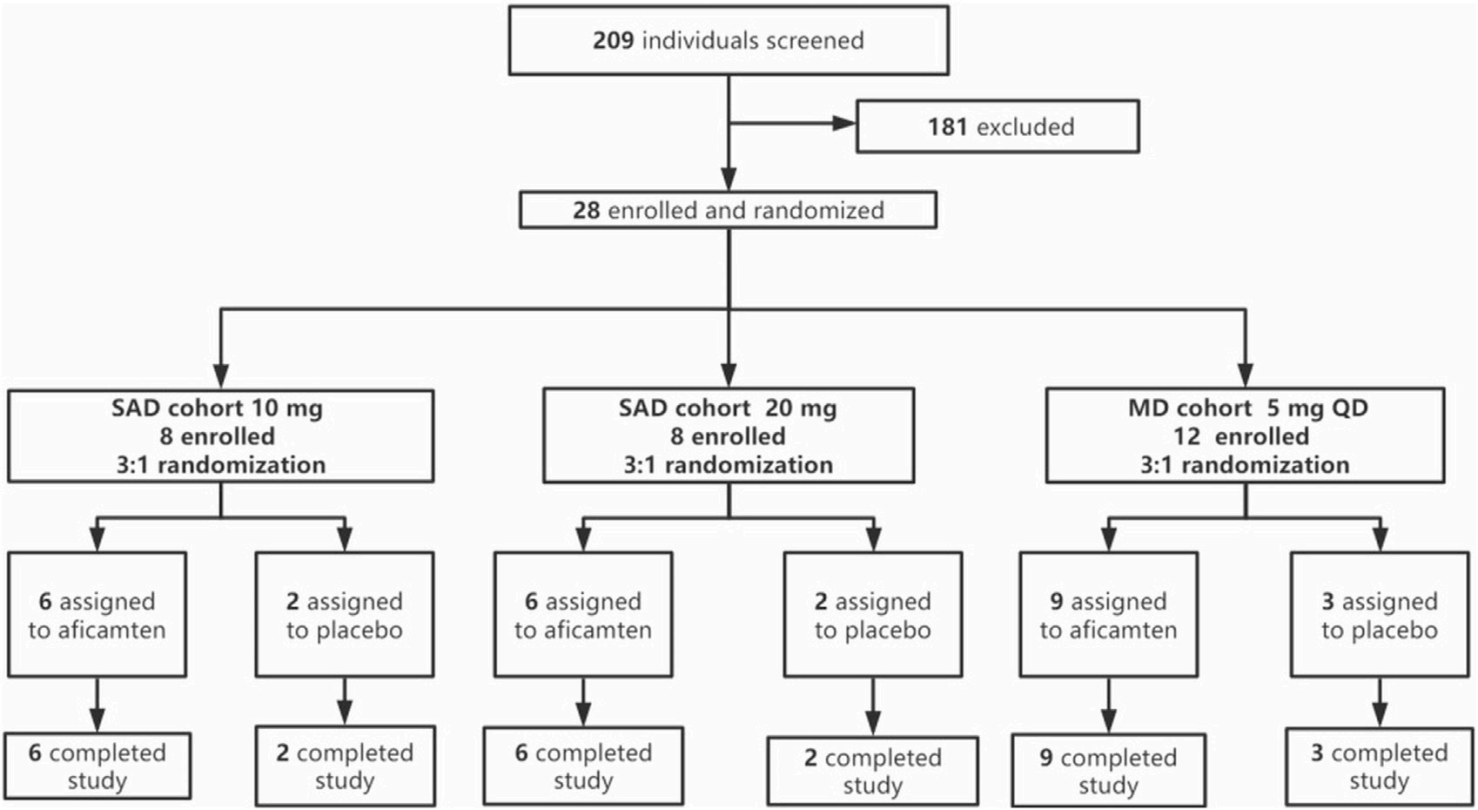
Trial profile.

This was a double-blind, randomized, placebo-controlled, phase 1 study including SAD and MD dose cohorts (Figure 1). As the tolerability and safety of aficamten were well-demonstrated by previous preclinical and clinical studies, a starting single dose of 10 mg was selected in the current study. For the second single-dose cohort, 20 mg was chosen as the escalated dose according to the dosing plan scheduled in the phase 3 study (CY 6031: NCT05186818). Furthermore, a multiple oral dose of 5 mg once daily (QD) was selected to explore the steady-state PK exposure in Chinese participants and to compare the findings with the MAD 5 mg QD results in the study CY 6011.

Of note, the study was not designed to identify a maximum tolerated dose. Dose escalation was to stop if a non-tolerated dose was identified. PD endpoints, including left ventricular ejection fraction (LVEF), would be measured with echocardiograms, which were reviewed by an independent cardiologist for safety assessments. The study drug would be discontinued for participants who develop left ventricular dysfunction according to pre-specified stopping rules. For additional safety monitoring, an independent, unblinded cardiologist was present at the clinical research unit from dosing through the completion of the echocardiogram on the last dosing day.

A total of 28 participants were enrolled in the study, with eight participants per SAD cohort (10 mg and 20 mg) and 12 participants in the MD cohort (5 mg). The sample size chosen for this study was based on other precedent PK studies of similar nature and was not based on power calculations. In each SAD cohort, eight eligible participants were randomized in 3:1 ratio to receive a single oral dose of aficamten or the matching placebo after overnight fasting. The dose escalation criteria (see Supplementary File S1) had to be met to proceed from the SAD 10 mg cohort to the SAD 20 mg cohort. In addition, the SAD 20 mg cohort started at least 5 days after the last subject’s administration in the SAD 10 mg cohort. Upon the completion of the SAD 20 mg cohort, the study proceeded to the MD 5 mg cohort when the dose escalation criteria were met. The MD cohort started at least 5 days later after the last subject’s administration in the SAD 20 mg cohort. In the MD cohort, 12 eligible participants were enrolled and randomized in 3:1 ratio to receive multiple oral doses of 5 mg of aficamten or the matching placebo under fasting conditions for 14 days. The investigator would determine if stopping rules (see Supplementary File S2) were met in this cohort.

### 2.2 Participants

In preclinical studies, high exposures of aficamten were associated with left ventricular systolic dysfunction, which is the intended pharmacological effect of this mechanism of action. The participants were required to meet specified LVEF requirements to be included in this study, and the left ventricular function was monitored closely during the study using an echocardiogram.

Potential participants were screened within 28 days prior to day 1 to assess their eligibility to enter the study. The key inclusion criteria were as follows: previously healthy male and female Chinese adults aged 18–45 years old (inclusive); body weight ≥50 kg; a body mass index between 18 and 26 kg/m^2^ (inclusive); normal cardiac structure and function; normal electrocardiogram (ECG); left ventricular ejection fraction (LVEF) ≥65% at screening; and LVEF ≥60% at day 1. The key exclusion criteria were as follows: significant history of prior system illness; active drug abuse or smoking; hypersensitivity to study compounds; known altered gastrointestinal absorption; recent enrollment in a competing study; and recent use of over-the-counter medications or blood donation. Participants were free to withdraw from the study at any time and for any reason. Details of inclusion and exclusion criteria are provided in Supplementary Files 3, 4.

### 2.3 Sample collection and analysis

Blood samples (4 mL) for plasma PK analysis of aficamten and its metabolites (mono-hydroxylated derivative CK-3834282 and its diastereomer CK-3834283) were collected into K2EDTA tubes in the SAD cohorts at the following time points: at pre-dose and at 0.25, 0.5, 1, 1.5, 2, 2.5, 3, 4, 6, 8, 12, 16, 24, 36, 48, 72, 96, and 216 h post-dose. In the MD cohort, blood samples were collected at pre-dose and at 0.25, 0.5, 1, 1.5, 2, 2.5, 3, 4, 6, 8, 12, and 16 h post-dose on day 1; at pre-dose and at 1.5 h post-dose on day 2, days 4–6, and day 9; at pre-dose on day 3, days 7–8, and days 10–13; at pre-dose and at 0.25, 0.5, 1, 1.5, 2, 2.5, 3, 4, 6, 8, 12, 16, 24, 36, 48, 72, 168, and 216 h post-dose on day 14. After collection, blood samples were centrifuged at 1700 *g* for 10 min at 4°C ± 2°C; plasma was aliquoted and stored at −80°C ± 20°C until analyzed. Meanwhile, samples for urine PK analysis of aficamten were collected in the MD cohort as follows: pre-dose, 0–4 h, 4–8 h, 8–12 h, and 12–24 h on days 1 and 14, respectively.

The samples were determined using a validated ultra-performance liquid chromatography-tandem mass spectrometry (UPLC-MS/MS) method. The detection was performed on a tandem mass spectrometer in the electrospray ionization (ESI)-positive mode. The multiple reactions monitoring (MRM) mode was applied to monitor the following ion transitions: m/z 338.2→213.2 for aficamten and m/z 354.3→ 229.3 for metabolite CK-3834282 and its diastereomer CK-3834283. Note that the diastereomers were separated from chromatography during the analysis. The calibration curves of the plasma and urine samples were linear, ranging from 1 to 500 ng/mL, and the intra- and inter-run accuracies (RE%) and precisions (RSD%) were within ±15%. In addition, the selectivity, matrix effect, extraction recovery, carryover, and stability at different conditions were also fully evaluated.

### 2.4 Pharmacokinetic evaluation

The plasma PK parameters were evaluated using the plasma concentrations by the non-compartmental model approach using WinNonlin (version 8.3, Certara LP, Princeton, NJ, USA) and were summarized descriptively by the cohort and dose. For the SAD cohorts, PK parameters included, but were not limited to, the maximum observed concentration (C_max_), the time to reach C_max_ (t_max_), and the area under the concentration-time curve from time 0 to 24 h (AUC_24_), from time 0 to the last measurable plasma concentration (AUC_0-t_), and from time 0 extrapolated to infinity (AUC_0-∞_), the terminal elimination half-life (t_1/2_), apparent clearance after oral administration (CL/F), and apparent volume of distribution during the terminal phase after oral administration (V_z_/F). For the MD cohort, PK parameters included, but were not limited to, C_max_ (day 1), t_max_ (day 1), C_max_ at steady state (C_max,ss_, day 14), t_max_ at steady state (t_max,ss_, day 14), AUC_24_ (days 1 and 14), and t_1/2_ (day 14). The accumulation ratio (AR) from day 1 to day 14 was estimated for C_max_ (AR_C_max_) and AUC_0-24_ (AR_AUC_0-24_) following multiple dosing.

Pre-dose trough concentration points on day 2 to day 14 in the MD 5 mg cohort were logarithmically transformed using the Helmert transformation approach in analysis of variance (ANOVA), and then a steady-state analysis was performed with study day as the fixed effect. First, the trough concentration at the first point (day 2) was compared to the mean trough concentration of day 3 to day 14. In the case of *p* value ≤0.05, the trough concentration at the second time point (day 3) was compared to the mean trough concentration of day 4 to day 14 and so forth until the first time point with *p* value >0.05, i.e., the time to steady state. In addition, the dose proportionality of C_max_ and AUC_0-24_ was examined on day 1 over the dose range tested (5, 10, and 20 mg) using a power model: ln(y) = *α* + β×ln (dose), where y represented the AUC and C_max_.

The urine PK parameters (MD cohort) were calculated as follows: CLR (renal clearance), Ae (amount of drug excreted unchanged in the urine), and Fe (percentage of drug excreted unchanged in the urine).

### 2.5 CYP2D6 genotypes

As CYP2D6 was one of the major metabolic enzymes for aficamten, the purpose of this testing was to evaluate the effect of CYP2D6 on the metabolism of aficamten in the Chinese population. A blood sample was collected for each subject on day 1 pre-dose in SAD and MD dose cohorts. Then, next-generation sequencing was used to detect CYP2D6 genotypes.

### 2.6 Pharmacodynamic evaluation

Echocardiography assessments (left ventricular fractional shortening [LVFS], left ventricular stroke volume [LVSV], left ventricular outflow tract velocity time integral [LVOT-VTI], left ventricular end-systolic volume [LVESV], left ventricular end-diastolic volume [LVEDV], left ventricular cardiac output [LVCO], and LVEF) for the SAD cohorts were collected on day 1 (baseline) and at 1.5, 4, and 24 h post-dose on day 1. For the MD cohort, the assessments were collected on day 1 (baseline) at 1.5 and 4 h post-dose on day 1, at 1.5 h post-dose on days 2, 4, and 9, and at 3 and 24 h post-dose on day 14.

Of all the echocardiography parameters, the key parameters LVEF and LVFS were classified as follows with the number of participants summarized for each dosing condition: for the LVEF category, baseline LVEF ≥60%, and post-dose LVEF <50% or <45%; change of LVEF from baseline ≤ 5%, ≤10%, or ˂15%; for the VLFS category, change of LVFS from baseline ≤ 5%, ≤10%, or ˂15%. The dose–PD response relationship between the aficamten dose and placebo group was assessed using ANOVA analysis. The PD parameters were analyzed to compare whether the change of PD parameters in each dose group of SAD cohorts and MD cohort was significantly different from those in the placebo groups at multiple post-dose time points. Furthermore, the relationship between PK concentration–PD response was also assessed using ANCOVA analysis of post-dose PD parameters based on changes from baseline. The model included changes from baseline in PD parameters as the dependent variables, concentration tertiles (categorical variables) as fixed effects, baseline PD parameters as covariates, and the random intercept to account for repeated measures. In addition, the heart rate (HR), systolic blood pressure (SBP), and diastolic blood pressure (DBP) were collected as a part of the vital sign assessments at pre-dose and post-dose.

### 2.7 Safety assessments

Safety was evaluated based on the occurrence, frequency, and severity of adverse events (AEs). Routine clinical laboratory tests, 12‐lead ECGs, physical examinations, and vital signs (blood pressure, pulse rate, and body temperature) were performed at initial screening, day 1, during the treatment period until discharge or early termination. The AEs were monitored from the initial administration to the last follow-up after the last treatment. The AEs were assessed by the investigators with regard to severity (mild, moderate, severe, and life‐threatening) and the relationship to study treatment (reasonably or possibly related and not reasonably or not possibly related).

### 2.8 Statistical analysis

Statistical analysis was performed using SAS version 9.1.3 (SAS Institute, Cary, North Carolina). Data were analyzed overall and by treatment and dose levels for each study part separately. Descriptive statistics for continuous variables included numbers of participants, means, medians, standard deviations, minima, and maxima. For categorical variables, frequencies and percentages were given. The baseline was defined as the last available measurement taken before the first dose of study medication, unless otherwise specified. ANCOVA analysis was performed using a linear mixed-effect model with intended variables. The PK analysis set consisted of all participants administered aficamten or placebo with at least one evaluable PK plasma profile, provided they have no major protocol violations that could affect the PK properties. Correspondingly, the PD analysis set included all participants administered aficamten or placebo with at least one echocardiographic parameter.

## 3 Results

### 3.1 Participant disposition

Of a total of 209 individuals screened, 28 eligible participants were enrolled and randomized into the study, with 16 participants in the SAD cohorts and 12 participants in the MD cohort. The enrolled participants were randomized in a ratio of 3:1 (aficamten: placebo) in each SAD cohort (10 mg and 20 mg) and MD cohort (5 mg). All dose escalation criteria were met in the study when the dose was escalated from SAD 10 mg to SAD 20 mg and from SAD 20 mg to MD 5 mg. No stopping rules were met in the MD 5 mg cohort. All 28 enrolled participants completed the study and were included in the PK, PD, and safety analyses. Treatment compliance for both SAD and MD cohorts was 100%. Demographic and baseline descriptions for all participants by overall characteristics are summarized in Table 1.

**TABLE 1:** Demographics and baseline characteristics.

Variable/Category	Statistic	SAD 10 mg (N = 6)	SAD 20 mg (N = 6)	SAD placebo (N = 4)	MD 5 mg (N = 9)	MD placebo (N = 3)	Overall (N = 28)
Sex							
Male	n (%)	4 (66.7)	5 (83.3)	4 (100.0)	7 (77.8)	2 (66.7)	22 (78.6)
Female	n (%)	2 (33.3)	1 (16.7)	0	2 (22.2)	1 (33.3)	6 (21.4)
Race							
Chinese	n (%)	6 (100.0)	6 (100.0)	4 (100.0)	9 (100.0)	3 (100.0)	28 (100.0)
Non-Chinese	n (%)	0	0	0	0	0	0
Age (years)	Mean	28.8	31.7	31.5	33.2	32.0	31.6
	SD	6.68	3.56	9.26	6.61	7.81	6.34
Weight (kg)	Mean	65.12	71.02	67.53	67.90	63.93	67.49
	SD	10.734	8.939	9.662	7.357	5.953	8.405
BMI (kg/m^2^)	Mean	22.85	24.00	22.60	22.83	23.07	23.08
	SD	1.864	1.403	1.671	1.591	0.643	1.534
SBP (mmHg)	Mean	109.0	115.0	111.8	111.4	101.0	110.6
	SD	8.05	6.42	10.90	10.76	1.73	9.11
DBP (mmHg)	Mean	66.5	71.5	76.5	70.3	65.7	70.1
	SD	6.44	4.85	1.73	9.89	6.81	7.51
Heart rate (beats/min)	Mean	63.7	61.5	65.0	64.7	66.7	64.0
	SD	3.72	3.94	6.88	4.03	4.16	4.39
LVEF (%)	Mean	71.3	70.8	69.0	68.0	69.0	69.6
	SD	2.07	2.86	4.55	4.00	1.00	3.37

Note: BMI, body mass index; DBP, diastolic blood pressure; LVEF, left ventricular ejection fraction; MD, multiple doses; SAD, single ascending dose; SBP, systolic blood pressure.

### 3.2 Pharmacokinetics

**FIGURE 2:**
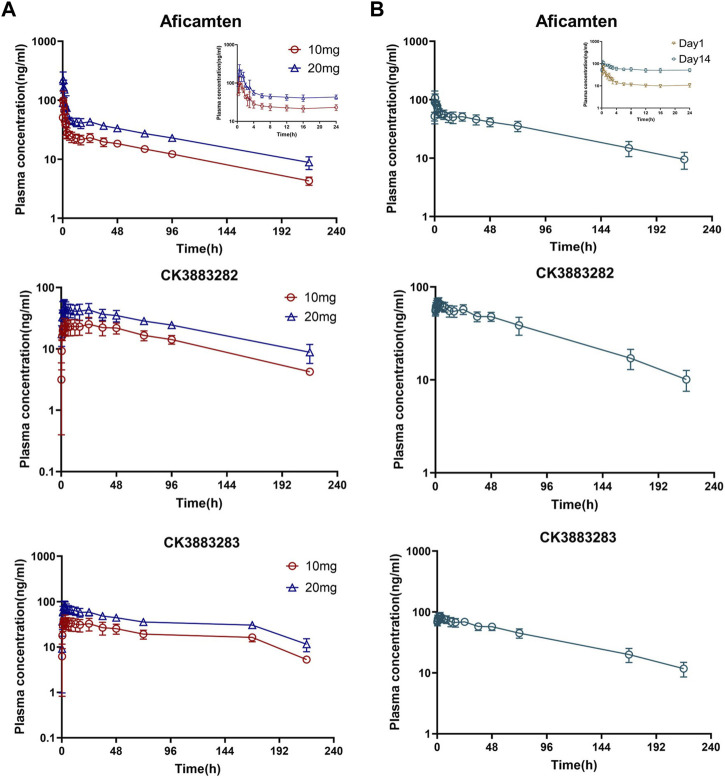
Plasma concentration–time profiles of aficamten and its metabolites in healthy Chinese participants. **(A)** SAD cohorts; **(B)** MD cohort.

The major plasma PK parameters of SAD and MD cohorts are summarized in Table 2. Following the single oral dose of 10 mg and 20 mg of aficamten in healthy Chinese participants, the concentration–time profiles of aficamten and metabolites (CK-3834282 and CK-3834283) in plasma were well-characterized (Figure 2A). For aficamten, peak plasma concentrations (C_max_) were reached at 0.5–0.75 h post-dose. In general, there was an initial rapid decline in PK, followed by a longer elimination phase after reaching peak concentration. The arithmetic mean t_1/2_ of aficamten was similar between 10 mg and 20 mg doses, ranging between approximately 74 and 77 h. For metabolites CK-3834282 and CK-3834283, peak concentration of either of the metabolites was reached at about 2.5 h, and the concentration gradually declined after the peak was reached with t_1/2_ of 82 h and 85 h, respectively. The PK exposure (AUC and C_max_) of aficamten and metabolites was increased with dose escalation. Following the administration of multiple oral doses of 5 mg of aficamten for 14 days in healthy Chinese participants, for aficamten, the mean plasma C_max_ was 72.6 ng/mL on day 1 and 121.9 ng/mL on day 14, respectively. The time periods to reach the peak concentration on day 1 and day 14 (median t_max,ss_) were the same: both were at 0.5 h. After reaching the peak concentration of aficamten, there was an initial rapid decline, followed by an elimination phase (Figure 2B). The arithmetic mean of t_1/2_ for aficamten on day 14 of the MD 5 mg cohort was approximately 77 h. The steady-state was reached on day 9 following the oral dosing of 5 mg once daily. In addition, the mean accumulation ratio of aficamten based on the overall exposure (AUC_0-24_) and that based on the peak exposure (C_max_) was 4.2 and 1.8, respectively. For metabolites CK-3834282 and CK-3834283, the mean accumulation ratio was 5.7 and 4.8 based on AUC_0-24_ and 5.9 and 4.4 based on C_max_, respectively. Following the multiple doses of 5 mg of aficamten in healthy Chinese participants, about 0.37% of the drug was excreted with urine in unchanged form, indicating that renal excretion is not the primary elimination route of aficamten.

**TABLE 2:** Pharmacokinetic parameters of aficamten and metabolites (CK-3834282 and CK-3834283) in healthy Chinese participants following single and multiple doses.

	PK parameters	SAD 10 mg (N = 6)	SAD 20 mg (N = 6)	MD 5 mg/day 1 (N = 9)	MD 5 mg/day 14 (N = 9)
Aficamten	AUC_0-24_ (h·ng/mL)	670.4 ± 85.0	1298.0 ± 151.2	325.3 ± 32.6	1356.0 ± 215.0
	AUC_0-t_ (h·ng/mL)	2783.0 ± 248.6	5294.0 ± 372.2	324.5 ± 32.5	6249 .0 ± 1243.0
	AUC_0-∞_ (h·ng/mL)	3337.0 ± 223.2	5989.0 ± 415.1	−	−
	C_max_^*^(ng/mL)	115.3 ± 29.9	227.3 ± 71.8	72.6 ± 19.5	121.9 ± 35.2
	t_1/2_ (h)	73.8 ± 10.2	77.3 ± 13.6	38.6 ± 16.1	76.8 ± 11.8
	t_max_^*^ (h)	0.75 (0.25, 3.0)	0.5 (0.5, 1.5)	0.5 (0.25, 1.00)	0.5 (0.25, 2.0)
	CL/F (L/h)	3.0 ± 0.19	3.4 ± 0.2	−	3.8 ± 0.6
	V/F (L)	320.3 ± 51.3	371.9 ± 55.4		412.7 ± 66.1
	AR_AUC	−	−	−	4.2 ± 0.4
	AR_C_max_	−	−	−	1.8 ± 0.6
	CL_r_ (mL/h)	−	−	9.5 ± 6.4	13.7 ± 5.8
	Ae_(0–24)_ (µg)	−	−	3.2 ± 2.6	18.5 ± 8.0
CK-3834282	AUC_0-24_ (h·ng/mL)	540.1 ± 145.4	991.0 ± 268.0	240.8 ± 47.8	1404.0 ± 171.0)
	AUC_0-t_ (h·ng/mL)	2901.0 ± 522.6	5107.0 ± 626.2	246.0 ± 41.4	6801.0 ± 744.1
	AUC_0-∞_ (h·ng/mL)	2901.0 ± 522.6	5107.0 ± 626.2	−	−
	C_max_^*^(ng/mL)	25.5 ± 7.6	49.3 ± 15.0	11.9 ± 1.90	69.4 ± 9.1
	t_1/2_ (h)	73.5 ± 11.0	89.9 ± 29.7	213.0 ± 117.8	78.0 ± 11.5
	t_max_^*^ (h)	18.0 (1.5, 24.0)	2.5 (1.5, 3.0)	3 (0.5, 23.9)	
	AR_AUC	−	−	−	5.7 ± 0.7
	AR_C_max_	−	−	−	5.9 ± 0.5
CK-3834283	AUC_0-24_ (h·ng/mL)	764.0 ± 224.4	1491.0 ± 315.3	367.7 ± 62.1	1735.0 ± 238.7
	AUC_0-t_ (h·ng/mL)	3555.0 ± 764.3	6749.0 ± 513.1	366.5 ± 61.9	8092.0 ± 1047.0
	AUC_0-∞_ (h·ng/mL)	3555.0 ± 764.3	6749.0 ± 513.1	−	−
	C_max_^*^(ng/mL)	39.5 ± 12.4	82.0 ± 21.8	20.2 ± 3.4	88.3 ± 13.5
	t_1/2_ (h)	79.1 ± 15.4	91.5 ± 26.7	79.2 ± 42.4	75.8 ± 9.6
	t_max_^*^ (h)	2.75 (1.50–3.00)	2.50 (1.50–3.00)	2.5 (0.5–3)	2.5 (1.5–3)
	AR_AUC	−	−	−	4.8 ± 0.4
	AR_C_max_	−	−	−	4.4 ± 0.5

PK, parameters are presented as arithmetic mean ± SD; SAD, single ascending dose; MD, multiple doses.

^*^: t_max_ values were presented as median (minimum, maximum).

^*^: C_max,ss_ and t_max,ss_ for day 14 of the MD 5 mg cohort.

Furthermore, following the first-day dosing of 5 mg and the single oral doses of 10 mg and 20 mg of aficamten, dose-proportionality was assessed using power model analysis. The point estimate and 90% CI for the ratio of dose‐normalized, geometric mean values of AUC_0-24_ and C_max_ were 0.996 (0.884, 1.134) and 0.769 (0.555, 1.06), respectively.

### 3.3 CYP2D6 genotypes

There was no poor metabolizer among all the participants dosed in the SAD cohorts and MD cohort, i.e., all participants were intermediate metabolizers (six subjects in the SAD cohort; two participants in the MD cohort) or extensive metabolizers (10 subjects in the SAD cohort; 10 in the MD cohort).

### 3.4 Pharmacodynamics

**FIGURE 3:**
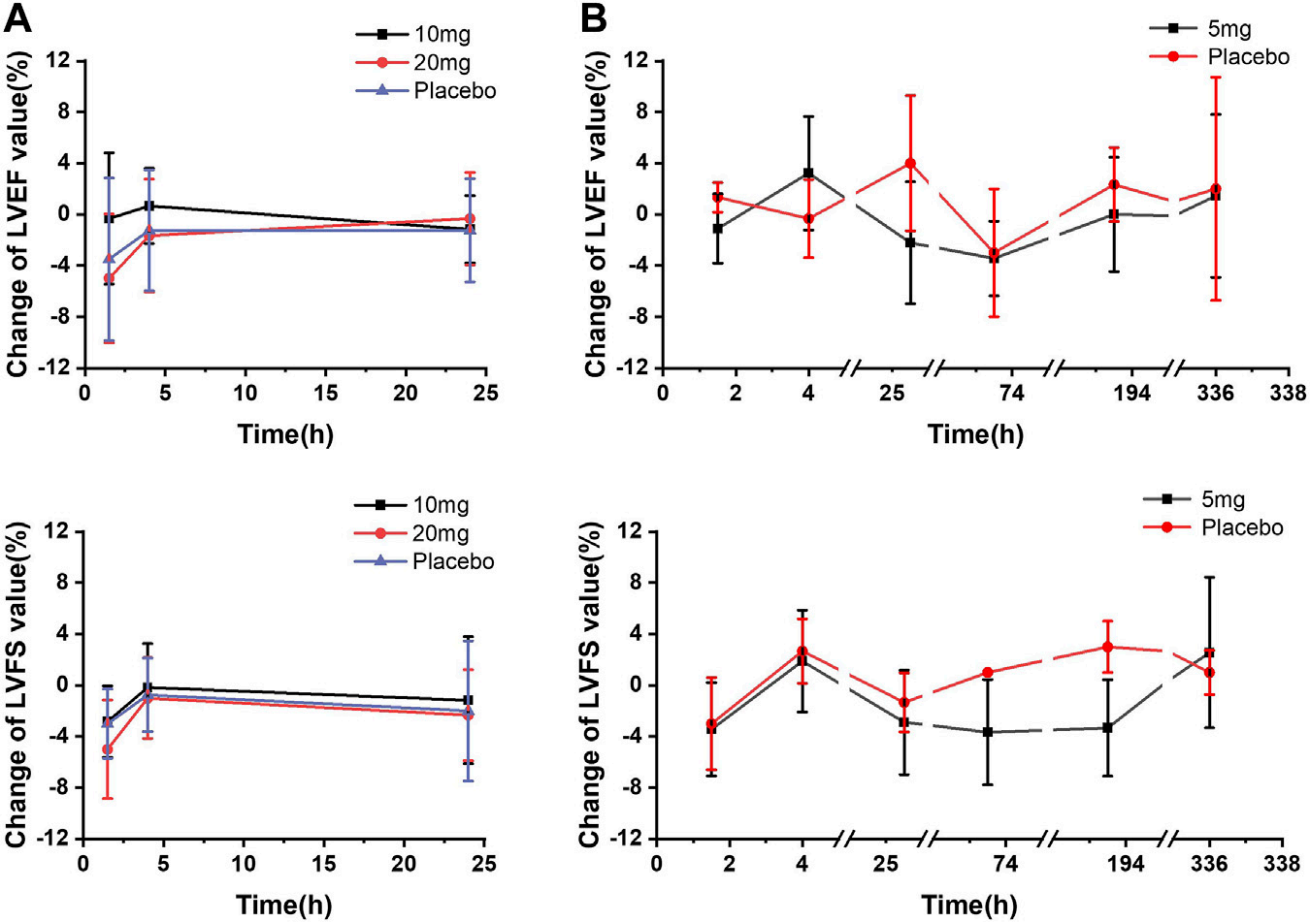
Mean echocardiographic PD parameter changes from baseline–time curves. **(A)** SAD cohorts; **(B)** MD cohort.

For echocardiography parameters (LVEF, LVFS, and LVOT-VTI), mean changes from baseline for PD parameter–time profiles were plotted and presented in Figure 3. Changes from baseline in LVEF and LVFS were summarized according to the following categories: change ≤5%, change ≤10%, and change < 15%. According to the LVEF measurement results, neither LVEF <50% nor LVEF reduction >15% was observed. Generally, moderate changes ≤ 5% from baseline in LVEF and LVFS were observed for all dose groups, including the placebo group (detailed data presented in Supplementary File; Supplementary Tables S1, S2).

In the SAD cohorts, change ≤ 5% from baseline in LVEF was observed in each dose group. Among these participants, LVEF reduction of 5%–10% occurred in one subject in the 10 mg-dose group and three participants in the 20 mg-dose group 1.5 h post-dose, and one subject with the LVEF reduction of 10%–15% was also observed in the 20 mg group 1.5 h post-dose. In the MD cohort, no significant difference was found in the number of participants, with reductions between day 1 and day 14 following multiple administrations of 5 mg. Overall, there was no statistically significant difference between any dose group and the placebo group in terms of PD parameter changes from baseline. LVEF results showed statistical differences in exposure and response in the PK concentration–PD effect analysis.

### 3.5 Exposure–response analysis

**FIGURE 4:**
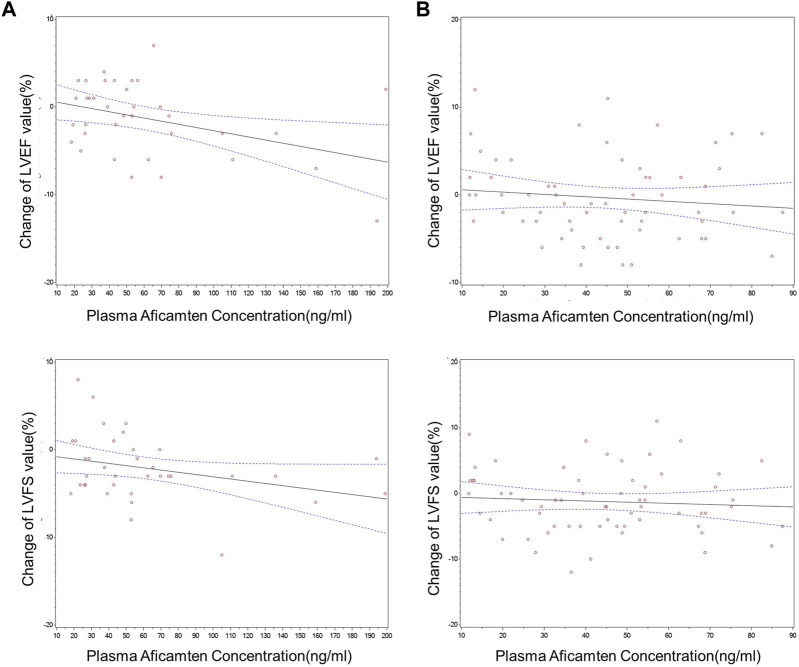
Changes from baseline in echocardiographic PD parameters vs. time-matched plasma aficamten concentrations. **(A)** SAD cohorts; **(B)** MD cohort.

Changes from baseline in echocardiography parameters increased mildly as concentrations of aficamten increased (Figure 4). For the SAD cohorts, significant inverse PK–PD relationships were observed for plasma concentrations of aficamten and LVEF and LVFS. After grouping by PK concentrations (group 1: 18.1–37.0 ng/mL; group 2: 37.6–56.3 ng/mL; group 3: 62.5–199.0 ng/mL), statistically significant changes from baseline were observed in LVEF results in group 3 and LVFS results in group 2 as compared with the placebo group (*p* < 0.05), respectively. Overall, an exposure–response analysis of change from baseline in LVEF and LVFS and time-matched PK concentrations showed a nominally statistically significant (*p* < 0.05) concentration effect.

However, for the MD 5 mg cohort, overall exposure–response analysis of change from baseline in echocardiogram parameters and time-matched plasma aficamten concentrations did not show a statistically significant effect for any of the parameters. With grouping by PK concentration (group 1: 11.8–21.9 ng/mL; group 2: 24.7–36.5 ng/mL; group 3: 38.3–48.5 ng/mL), statistically significant difference of LVEF or LVFS was not observed in the comparison between the aficamten and placebo groups.

### 3.6 Safety

In healthy Chinese participants, aficamten was well-tolerated after oral administration of a single dose of 10 mg or 20 mg and multiple doses of 5 mg. There were no deaths, serious adverse events (SAEs), AE of special interests (AESIs), participants who discontinued due to treatment-emergent AEs (TEAEs), or LVEF <50%. Overall, a total of 35 TEAEs were reported by 14 (50%) participants (detailed data presented in Supplementary File Table S3). The most common TEAEs were those classified according to system organ class as investigations, with the TEAEs of systolic blood pressure decreased (6 participants, 21.4%), conjugated bilirubin increased (four participants, 14.3%), heart rate decreased (two participants, 7.1%), aspartate aminotransferase increased (two participants, 7.1%), and neutrophil count decreased (two participants, 7.1%) being reported in more than one subject each. While there were more reports of increased bilirubin in aficamten groups compared to the placebo group, the increases were small and rapidly returned to baseline. Other abnormal laboratory values and decreases in SBP were marginal, transient, asymptomatic, and comparable between the aficamten and placebo groups. All TEAEs were mild in severity. The majority (31 of 35) of TEAEs were considered by investigators as related to the investigational product. All TEAEs were transient, self-limiting, and resolved without sequelae. There were no clinically notable findings of a safety concern in the clinical safety laboratory tests, vital signs, ECGs, or echocardiogram parameters during this study.

## 4 Discussion

Aficamten is a selective and next-in-class cardiac myosin inhibitor (Chuang et al., 2021). With the supportive data of aficamten from preclinical and clinical studies in Western populations (CY 6011 and CY 6021), for the first time, aficamten was evaluated in healthy Chinese participants. The PK, PD, and safety data after the oral administration of single-dose and multi-dose administrations of aficamten in healthy Chinese participants were obtained in the study.

Given the same treatment regimen, the PK characteristics of aficamten in healthy Chinese participants were comparable to those in healthy Western participants. In healthy Chinese participants, the aficamten plasma exposures (AUC_0-24_ and C_max_) were found to be dose-proportional following the first-day dosing of MD 5 mg and the single oral doses of 10 mg and 20 mg. Similar dose-proportional overall exposures were reported in study CY 6011 in healthy Western participants (Robertson et al., 2019; Malik et al., 2022). In the SAD 10 mg cohort, the median t_max_ post-dose of healthy Chinese participants was slightly earlier (0.75 h) than that of healthy Western participants (1.8 h) (Malik et al., 2022). The mean (SD) of the overall exposures (AUC_0-∞_ and C_max_) of aficamten in Chinese and Western participants were 3337.0 (223.2) vs. 3434.0 (24.0) ng∙h/mL and 115.3 (29.9) vs. 70.5 (69.0) and ng/mL, respectively. In addition, the t_1/2_, CL/F, and V/F were also similar between healthy Chinese and Western participants. Following 14 days of QD oral doses of 5 mg of aficamten, the mean accumulation ratio based on the overall exposure (AR_AUC_0-24_) was 4.16 in healthy Chinese participants, which was comparable to the accumulation ratio of 4.71 reported for the healthy Western participants in CY 6011 for the same dose (Robertson et al., 2019; Malik et al., 2022). In healthy Chinese participants, aficamten plasma concentrations declined with a mean half-life of 74–77 h following a single oral dose of 10 mg and 20 mg and following 14 days of multiple QD oral doses of 5 mg. These results are consistent with the average half-life of 75–85 h for the healthy Western participants in the CY 6011 MAD cohorts ranging from 5 mg to 10 mg (Robertson et al., 2019; Malik et al., 2022). Steady-state conditions were attained within 9 days of repeated administration in healthy Chinese participants, which were similar to the results in CY 6011, where the steady-state appeared evident after 12–14 days of daily dosing in healthy Western participants (Robertson et al., 2019; Malik et al., 2022). Aficamten reached its peak concentration at 0.50–0.75 h in all cohorts (referred to as t_max_ on day 1 and t_max,ss_ on day 14) for healthy Chinese participants, which was earlier than but still close to the t_max_ for the healthy Western population in CY 6011, which ranged between 1.0 and 2.75 h.

The CYP polymorphism frequencies of CYP2D6 PM and intermediate metabolizers (IMs) vary significantly between Chinese and Caucasian populations. In the Chinese population, the CYP2D6 PM accounts for 0.3%, and the CYP2D6 IM accounts for 39%; In the Caucasian population, the CYP2D6 PM accounts for 8%, and the CYP2D6 IM accounts for less than 1% (Barter et al., 2013). In the current first-in-Chinese study, there was no poor metabolizer among all the participants dosed in the SAD cohorts and MD cohort. Although intrinsic factors significantly influence drug elimination, study data from CY 6011 showed that the exposure to aficamten was comparable in the CYP2D6-PM cohort and the cohort of non-poor metabolizer participants (Malik et al., 2022). These data suggested that ethnic differences in PK based on CYP2D6 genotype were not expected.

**FIGURE 5:**
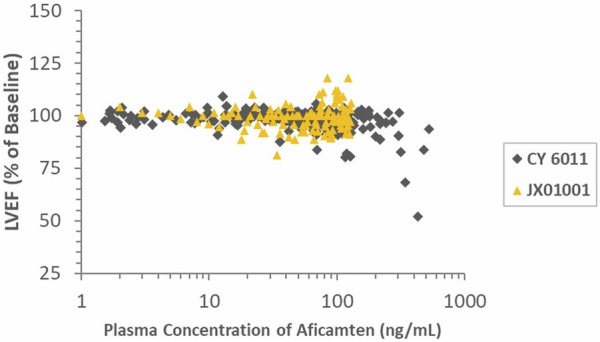
Ratios of LVEF and baseline in healthy Chinese and Western participants (study CY 6011) vs. time-matched plasma aficamten concentrations.

Significant inverse PK–PD relationships were observed for plasma concentrations of aficamten and LVEF and LVFS in the SAD groups but not in the MD group, which showed relatively lower concentrations and narrower concentration range compared to the SAD groups. The results should be interpreted with caution given the small sample size and narrow concentration range evaluated in this study. The ratios of LVEF and baseline in the heathy Chinese (JX01001) and Western participants (study CY 6021) are shown in Figure 5. In general, the LVEF-to-baseline ratios tend to decrease with increasing plasma concentrations, and the data of the healthy Chinese and healthy Western participants were highly overlapped, indicating that aficamten in Chinese participants was consistent with the effect on LVOT-G as described in Western participants.

In the current study, the safety and tolerability of aficamten were fully evaluated in the heathy Chinese participants after single and multiple doses of aficamten. Overall, the safety profile of aficamten was consistent between the healthy Chinese and the healthy Western participants, and aficamten was well-tolerated in these two populations. There were no deaths, SAEs, or discontinuations due to TEAEs. All TEAEs were mild in severity. There were no clinically significant findings of a safety concern in the laboratory tests, vital signs, ECGs, or echocardiogram parameters in this China phase 1 study or CY 6011 (Malik et al., 2022).

Direct inhibition of cardiac myosin to relieve hypercontraction and LVOT obstruction in patients with oHCM is a targeted pharmacological approach intended to improve functional capacity and symptoms. To this aim, target therapeutic potential has been demonstrated by mavacamten, another selective cardiac myosin inhibitor, in a phase 3 trial (Olivotto et al., 2020). Although mechanism of action is similar, the pharmacokinetic profile of aficamten differs from that of mavacamten in certain aspects. Mavacamten was reported to possess a half-life of 7–9 days in humans, and the steady-state was reached at approximately 6 weeks (Heitner et al., 2019; Olivotto et al., 2020). As a comparison, the much shorter half-life of aficamten potentially allows for more rapid dose titration and more rapid reversal of drug exposure by down-titration when necessary.

Based on the above data, it is evident that PK and safety profiles are consistent between healthy Chinese and Western population. Thus, aficamten could be advanced to the study of Chinese oHCM patients based on the readily available preliminary data of aficamten in Western patients (Robertson et al., 2019). Accordingly, China has joined the global study CY 6031 (SEQUOIA-HCM; NCT05186818), a phase 3, multi-center, randomized, double-blind, placebo-controlled study. In this study, the clinical efficacy and safety profiles of aficamten will be evaluated in patients with symptomatic oHCM and LVOT-gradient (LVOT-G) >50 mmHg post-Valsalva.

## 5 Conclusion

In conclusion, we designed and completed a first-in-Chinese phase 1 study of aficamten. The results demonstrated that aficamten was safe and well-tolerated at the doses studied in healthy Chinese adult participants. The PK parameters of aficamten in healthy Chinese participants were comparable to those previously found in healthy Western participants when administered at the same doses. The safety profile, PK, and PD results in healthy Chinese participants support the further evaluation of the efficacy and safety of aficamten in Chinese oHCM patients.

## Data Availability

The raw data supporting the conclusion of this article will be made available by the authors, without undue reservation.
