# Supplementary material for: Safety, tolerability, pharmacokinetics, and pharmacodynamics of single and multiple doses of aficamten in healthy Chinese participants: a randomized, double-blind, placebo-controlled, phase 1 study
Source: Front Pharmacol. 2023 Aug 23;14:1227470. doi: 10.3389/fphar.2023.1227470 (PMC10482267; doi:10.3389/fphar.2023.1227470)
Supplement: Supplementary file 1 [file Table1.DOCX]

Supplementary Material

Article Title

**Xue Zhao^1†^, Hongzhong Liu^1†^, Wei Tian^1^, Ligang Fang^2^, Mengyang Yu ^1^, Xiaofei Wu ^1^, Aijing Liu ^1^, Ruijie Wan^1^, Li Li^1^, Jinghui Luo^3^, Yuqiong Li^3^, Bo Liu^3^, Yu He^3^, Xiaowen Chen^3^, Yuan Li^3^, Donghong Xu^4^, Hongyun Wang^1^*, Xiaohong Han^1^***

^1^ Clinical Pharmacology Research Center, Peking Union Medical College Hospital, Beijing, P. R. China

^2^ Internal Medicine-Cardiovascular Department, Peking Union Medical College Hospital, Beijing, P. R. China

^3^ Ji Xing Pharmaceuticals (Shanghai) Co., Ltd., Shanghai, P. R. China

^4^ Cytokinetics, Incorporated, South San Francisco, CA, USA

**†Equal contribution and first authorship:** These authors contributed equally to this work and share first authorship.

*** Correspondence:**Hongyun Wang
[wanghy@pumch.cn](mailto:wanghy@pumch.cn);

Xiaohong Han
hanxiaohong@pumch.cn

- **File 1: Dose Escalation Criteria**
- **File 2: Stopping Rules**
- **File 3: Subject Inclusion Criteria**
- **File 4: Subject Exclusion Criteria**
- **Table S1.** Summary of All TEAEs by System Organ Class and Preferred Term
- **Table S2.** Categorical Summary for Left Ventricular Ejection Fraction (LVEF) and Left Ventricular Fractional Shortening (LVFS) Change From Baseline by Treatment (SAD cohorts)
- **Table S3.** Categorical Summary for LVEF and LVFS Change From Baseline by Treatment (MD cohort)

**File 1: Dose Escalation Criteria**

Dose escalation decisions were made jointly by the Investigator (blinded), independent cardiologist (unblinded), and Sponsor’s Medical Monitor (blinded) based on their clinical judgement after review of the available safety, tolerability data, and echocardiograms results. To escalate doses, all the following criteria were met:

- No subjects experienced a cardiac serious adverse event (SAE) related to study drug;
- No two subjects experienced similar, non-cardiac SAEs in the same organ system that appeared to be related to study drug;
- No two subjects treated with aficamten had a decrease in LVEF >15% from last pre-dose value (determined by the unblinded independent cardiologist);
- No more than two subjects had a LVEF <50% (unless determined not to be related to the study drug by the Investigator, independent cardiologist, and Sponsor’s Medical Monitor);
- No subject had a LVEF <45% (unless determined not to be related to the study drug by the Investigator, independent cardiologist, and Sponsor’s Medical Monitor);
- Other adverse events (AEs) were reviewed by the Investigator, independent cardiologist, and Sponsor’s Medical Monitor;
- The Investigator, independent cardiologist, and Sponsor’s Medical Monitor agreed on the escalation based on their clinical judgement.

**File 2: Stopping Rules**

Dosing within the MD 5 mg cohort would be stopped if one or more of the followings occurred:

- One or more subjects had a cardiac SAE related to study drug;
- Two or more subjects had related SAEs in the same organ class and related to study drug;
- Two or more subjects had a decrease in LVEF >15% from Day-1 value (determined by the unblinded independent cardiologist); Two or more subjects had a LVEF <50% (unless determined not to be related to the study drug by the Investigator, independent cardiologist, and Sponsor’s Medical Monitor);
- One or more subjects had an LVEF <45% (unless determined not to be related to the study drug by the Investigator, independent cardiologist, and Sponsor’s Medical Monitor);
- The Investigator, or independent cardiologist, or Sponsor’s Medical Monitor determined that dosing should pause for any other reason.

**File 3: Subject Inclusion Criteria**

Subjects who meet all the following criteria at Screening may be included in the study:

1. Able to comprehend and willing to sign an informed consent form (ICF) and willing to comply with all study procedures and restrictions including remaining in the CRU for the duration specified in the SoA
2. Males and females between 18 and 45 years of age, inclusive
3. Body weight ≥ 50kg and body mass index within 18 to 26 kg/m2, inclusive
4. In good health, in the opinion of the Investigator, as determined by the following:

- A physical examination at screening and medical history with no clinically significant abnormalities
- Not taking medications for the treatment of any chronic or episodic medical disease or condition
- Vital signs within the ranges below measured while the subject is supine after 3 minutes rest. May re-check vital signs twice at screening.

Heart rate (HR): 60-100 beats per minute; systolic blood pressure (SBP):90-140 mmHg; diastolic blood pressure (DBP): 50-90mmHg; respiration rate: <25 breaths per minute.

1. Acoustic windows adequate for accurate transthoracic echocardiograms
2. Normal cardiac structure and function, as determined by the cardiologist, or if abnormalities are present, the finding is not clinically significant as determined by the cardiologist
3. LVEF ≥65% at screening, and LVEF ≥60% at Day-1 (admission to the CRU)
4. Normal ECG or, if abnormalities are present, they are deemed not clinically significant by the Investigator and if requested, Sponsor’s Medical Monitor
5. Clinical laboratory findings within normal range at Screening and admission to the CRU, or if outside of the normal range, are deemed not clinically significant by the Investigator, including:

- Troponin I ≤the upper limit of normal
- Hemoglobin ≥12.0 g/dL for males and ≥11.0 g/dL for females

1. Negative hepatitis panel (including hepatitis B surface antigen and hepatitis C antibody), negative human immunodeficiency virus antibody screens, and negative syphilis test at screening
2. Willing and able to refrain from strenuous exercise (e.g. activity which could be expected to cause muscle soreness) from 48 hours prior to Day-1 and during the period of confinement at the CRU, and willing to otherwise maintain a normal level of non- strenuous physical activity throughout the entire study
3. Male subjects who have not had a vasectomy must agree, for the duration of their participation in the study until 90 days following Study Completion, to abstain from sexual intercourse or use a condom and spermicide during sexual intercourse with female partners who are of childbearing potential AND to have female partners use one of the highly effective means of contraception as described below,

- Combined (estrogen and progestogen containing) oral, intravaginal, or transdermal hormonal contraception associated with inhibition of ovulation
- Progestogen-only oral, injectable, or implantable hormonal contraception associated with inhibition of ovulation
- Intrauterine device
- Intrauterine hormone-releasing system

1. Males must agree to refrain from sperm donation from admission to the CRU until 90 days following Study Completion
2. Female subjects of childbearing potential must not be pregnant (i.e. The results from a serum pregnancy test at screening and at Day-1 must be within the normal range) or lactating and must agree to use highly effective contraception (as described in Criteria12) or abstinence during their participation in the study until 90 days following Study Completion. Women with an intact uterus are deemed postmenopausal if they are at least age 45, have had cessation of menses for at least 1 year, and have not taken hormones or oral contraceptives (including estrogen or hormone replacement therapy) during the past 12 months. Postmenopausal women participating in the study will not be required to use contraception

**File 4: Subject Exclusion Criteria**

Any of the following will exclude potential subjects from the study:

1. Significant history or clinical manifestation of any significant metabolic, allergic/ immunologic, dermatological, hepatic, renal, hematological, pulmonary, cardiovascular (including arrhythmia), gastrointestinal, gallbladder/biliary, musculoskeletal, neurological, or psychiatric disorder
2. History of significant hypersensitivity or allergy to any drug compound or other substance, unless approved by the Investigator and the Sponsor’s Medical Monitor
3. History of stomach or intestinal surgery or resection that would potentially alter absorption and/or excretion of orally administered drugs (appendectomy, hernia repair, and/or cholecystectomy will be allowed)
4. Subjects with breast implants that may impede echocardiography
5. A clinically significant illness within 4 weeks prior to admission to the CRU
6. Inability to swallow tablets
7. History of or current substance abuse (drug or alcohol), known drug or alcohol dependence within the last 2 years prior to Screening, or positive test for drugs of abuse/ alcohol breath testing at the screening visit or on Day-1
8. Use of any tobacco-containing or nicotine-containing products (including but not limited to cigarettes, pipes, vape pens, hookahs, cigars, chewing tobacco, nicotine patches, nicotine lozenges, or nicotine gum) or history of use of daily tobacco within 3 months prior to admission to the CRU
9. Participation in any other investigational study drug trial in which receipt of an investigational study drug occurred within 30 days or 5 half-lives (whichever is longer) prior to admission to the CRU
10. Use of any prescription medications within 14 days or over-the-counter (OTC) medication within 7 days prior to admission to the CRU (excluding acetaminophen), or intent to use any prescription or OTC medication during the study that may interfere with the evaluation of the study drug
11. Use of any OTC, non-prescription preparations (including vitamins, minerals, and phytotherapeutic/herbal/plant-derived preparations) within 7 days prior to admission to the CRU, except for acetaminophen which are allowed up to 48 hours prior to admission to the CRU
12. Use of alcohol-containing, grapefruit-containing, or caffeine-containing foods or beverages within 48 hours prior to admission to the CRU
13. Poor peripheral venous access
14. Any blood donation within 60 days prior to admission to the CRU or any plasma donation within 30 days prior to admission to the CRU, or receipt of blood products within 2 months prior to admission to the CRU
15. Any acute or chronic condition that would limit the subject's ability to complete and/or participate in this clinical study, in the judgment of the Investigator
16. History, or suspicion, of inability to comply fully with all procedural aspects of the study
17. Involvement in the planning and/or conduct of the study (applies to both Sponsor staff and/or staff at the study site)

**Table S1. Summary of all TEAEs by system organ class and preferred term**

|  | | **Number (%) of Subjects /Events** | | | | | |
| --- | --- | --- | --- | --- | --- | --- | --- |
| **System Organ Class**  **Preferred Term** |  | **SAD 10 mg**  **(N = 6)** | **SAD 20 mg**  **(N = 6)** | **SAD Placebo**  **(N = 4)** | **MD 5 mg**  **(N = 9)** | **MD Placebo**  **(N = 3)** | **Overall**  **(N = 28)** |
| TEAEs |  | 1(16.7) /3 | 4(66.7) /10 | 1(25.0) /4 | 6(66.7) /14 | 2(66.7) /4 | 14(50.0) /35 |
|  | | | | | | | |
| Investigations |  | 1(16.7) /2 | 4(66.7) /7 | 1(25.0) /4 | 6(66.7) /13 | 2(66.7) /4 | 14(50.0) /30 |
| Systolic blood pressure decreased |  | 0 | 2(33.3) /2 | 1(25.0) /1 | 2(22.2) /3 | 1(33.3) /1 | 6(21.4) /7 |
| Conjugated bilirubin increased |  | 0 | 2(33.3) /2 | 0 | 2(22.2) /2 | 0 | 4(14.3) /4 |
| Neutrophil count increased |  | 0 | 0 | 1(25.0) /1 | 1(11.1) /1 | 0 | 2( 7.1) /2 |
| Heart rate decreased |  | 0 | 1(16.7) /2 | 0 | 0 | 1(33.3) /3 | 2( 7.1) /5 |
| Neutrophil count decreased |  | 1(16.7) /1 | 0 | 0 | 1(11.1) /2 | 0 | 2( 7.1) /3 |
| Alanine aminotransferase increased |  | 0 | 0 | 1(25.0) /1 | 0 | 0 | 1( 3.6) /1 |
| Blood bilirubin increased |  | 0 | 1(16.7) /1 | 0 | 0 | 0 | 1( 3.6) /1 |
| Blood creatine phosphokinase increased |  | 0 | 0 | 0 | 1(11.1) /1 | 0 | 1( 3.6) /1 |
| Blood creatinine increased |  | 0 | 0 | 0 | 1(11.1) /1 | 0 | 1( 3.6) /1 |
| Blood sodium decreased |  | 0 | 0 | 0 | 1(11.1) /1 | 0 | 1( 3.6) /1 |
| Gamma-glutamyltransferase increased |  | 0 | 0 | 1(25.0) /1 | 0 | 0 | 1( 3.6) /1 |
| Neutrophil count increased |  | 0 | 0 | 0 | 1(11.1) /1 | 0 | 1( 3.6) /1 |
| White blood cell count decreased |  | 1(16.7) /1 | 0 | 0 | 0 | 0 | 1( 3.6) /1 |
| White blood cell count increased |  | 0 | 0 | 0 | 1(11.1) /1 | 0 | 1( 3.6) /1 |
|  | | | | | | | |
| Cardiac disorders |  | 1(16.7) /1 | 0 | 0 | 0 | 0 | 1( 3.6) /1 |
| Ventricular hypokinesia |  | 1(16.7) /1 | 0 | 0 | 0 | 0 | 1( 3.6) /1 |
|  | | | | | | | |
| Gastrointestinal disorders |  | 0 | 1(16.7) /1 | 0 | 0 | 0 | 1( 3.6) /1 |
| Nausea |  | 0 | 1(16.7) /1 | 0 | 0 | 0 | 1( 3.6) /1 |
|  | | | | | | | |
| General disorders and administration site conditions |  | 0 | 1(16.7) /2 | 0 | 0 | 0 | 1( 3.6) /2 |
| Chest discomfort |  | 0 | 1(16.7) /2 | 0 | 0 | 0 | 1( 3.6) /2 |
|  | | | | | | | |
| Skin and subcutaneous tissue disorders |  | 0 | 0 | 0 | 1(11.1) /1 | 0 | 1( 3.6) /1 |
| Rash |  | 0 | 0 | 0 | 1(11.1) /1 | 0 | 1( 3.6) /1 |

Notes: MedDRA = Medical Dictionary for Regulatory Activities; TEAE = treatment-emergent adverse event.

System organ class and preferred term are from the MedDRA version 24.0; MD = one multiple dose; SAD = single ascending dose.

Subjects with multiple events in the same category are counted only once in that category. Subjects with events in more than one category are counted once in each of those categories.

**Table S2. Categorical summary for left ventricular ejection fraction (LVEF) and left ventricular fractional shortening (LVFS) change from baseline by treatment (SAD cohorts)**

| **Time Point (Hour)** | **Category** | **Dose** | | |
| --- | --- | --- | --- | --- |
|  | **LVEF Change From Baseline** | **SAD 10 mg**  **N=6**  **n (%)** | **SAD 20 mg**  **N=6**  **n (%)** | **Placebo**  **N=4**  **n (%)** |
| 1.5 | ≤ -5 | 1 (16.67%) | 3 (50%) | 1 (25%) |
|  | ≤ -10 | 0 (0%) | 1 (16.67%) | 1 (25%) |
|  | ≤ -15 | 0 (0%) | 0 (0%) | 0 (0%) |
| 4 | ≤ -5 | 1 (16.67%) | 2 (33.33%) | 1 (25%) |
|  | ≤ -10 | 0 (0%) | 0 (0%) | 0 (0%) |
|  | ≤ -15 | 0 (0%) | 0 (0%) | 0 (0%) |
| 24 | ≤ -5 | 0 (0%) | 1 (16.67%) | 1 (25%) |
|  | ≤ -10 | 0 (0%) | 0 (0%) | 0 (0%) |
|  | ≤ -15 | 0 (0%) | 0 (0%) | 0 (0%) |

| **Time Point (Hour)** | **Category** | **Dose** | | |
| --- | --- | --- | --- | --- |
|  | **LVFS Change From Baseline** | **SAD 10 mg**  **N=6**  **n (%)** | **SAD 20 mg**  **N=6**  **n (%)** | **Placebo**  **N=4**  **n (%)** |
| 1.5 | ≤ -5 | 1 (16.67%) | 3 (50%) | 1 (25%) |
|  | ≤ -10 | 0 (0%) | 1 (16.67%) | 0 (0%) |
|  | ≤ -15 | 0 (0%) | 0 (0%) | 0 (0%) |
| 4 | ≤ -5 | 0 (0%) | 1 (16.67%) | 0 (0%) |
|  | ≤ -10 | 0 (0%) | 0 (0%) | 0 (0%) |
|  | ≤ -15 | 0 (0%) | 0 (0%) | 0 (0%) |
| 24 | ≤ -5 | 1 (16.67%) | 2 (33.33%) | 2 (50%) |
|  | ≤ -10 | 0 (0%) | 0 (0%) | 0 (0%) |
|  | ≤ -15 | 0 (0%) | 0 (0%) | 0 (0%) |

Baseline is defined as the last measurement prior to Day 1 dosing. Change from baseline is the absolute difference between the measured value at each time point after drug administration and the baseline value.

**Table S3. Categorical summary for left ventricular ejection fraction (LVEF) and left ventricular fractional shortening (LVFS) change from baseline by treatment (MD cohort)**

| **Time Point (Hour)** | **Category** | **Dose** | |
| --- | --- | --- | --- |
|  | **LVEF Change From Baseline** | **MD 5 mg**  **N=9**  **n (%)** | **Placebo**  **N=3**  **n (%)** |
| 1.5 | ≤ -5 | 1 (11.11%) | 0 (0%) |
|  | ≤ -10 | 0 (0%) | 0 (0%) |
|  | ≤ -15 | 0 (0%) | 0 (0%) |
| 25.5  (1.5 hours post-dose on Day 2) | ≤ -5 | 4 (44.44%) | 0 (0%) |
|  | ≤ -10 | 0 (0%) | 0 (0%) |
|  | ≤ -15 | 0 (0%) | 0 (0%) |
| 73.5  (1.5 hours post-dose on Day 4) | ≤ -5 | 3 (33.33%) | 1 (33.33%) |
|  | ≤ -10 | 0 (0%) | 0 (0%) |
|  | ≤ -15 | 0 (0%) | 0 (0%) |
| 193.5  (1.5 hours post-dose on Day 9) | ≤ -5 | 1 (11.11%) | 0 (0%) |
|  | ≤ -10 | 0 (0%) | 0 (0%) |
|  | ≤ -15 | 0 (0%) | 0 (0%) |
| 315  (3 hours post-dose on Day 14) | ≤ -5 | 2 (22.22%) | 0 (0%) |
|  | ≤ -10 | 0 (0%) | 0 (0%) |
|  | ≤ -15 | 0 (0%) | 0 (0%) |
| 336  (24 hours post-dose on Day 14) | ≤ -5 | 2 (22.22%) | 1 (33.33%) |
|  | ≤ -10 | 0 (0%) | 0 (0%) |
|  | ≤ -15 | 0 (0%) | 0 (0%) |

| **Time Point (Hour)** | **Category** | **Dose** | |
| --- | --- | --- | --- |
|  | **LVFS Change from Baseline** | **MD 5 mg**  **N=9**  **n (%)** | **Placebo**  **N=3**  **n (%)** |
| 1.5 | ≤ -5 | 3 (33.33%) | 1 (33.33%) |
|  | ≤ -10 | 1 (11.11%) | 0 (0%) |
|  | ≤ -15 | 0 (0%) | 0 (0%) |
| 25.5  (1.5 hours post-dose on Day 2) | ≤ -5 | 4 (44.44%) | 0 (0%) |
|  | ≤ -10 | 0 (0%) | 0 (0%) |
|  | ≤ -15 | 0 (0%) | 0 (0%) |
| 73.5  (1.5 hours post-dose on Day 4) | ≤ -5 | 5 (55.56%) | 0 (0%) |
|  | ≤ -10 | 1 (11.11%) | 0 (0%) |
|  | ≤ -15 | 0 (0%) | 0 (0%) |
| 193.5  (1.5 hours post-dose on Day 9) | ≤ -5 | 4 (44.44%) | 0 (0%) |
|  | ≤ -10 | 0 (0%) | 0 (0%) |
|  | ≤ -15 | 0 (0%) | 0 (0%) |
| 315  (3 hours post-dose on Day 14) | ≤ -5 | 1 (11.11%) | 0 (0%) |
|  | ≤ -10 | 0 (0%) | 0 (0%) |
|  | ≤ -15 | 0 (0%) | 0 (0%) |
| 336  (24 hours post-dose on Day 14) | ≤ -5 | 1 (11.11%) | 0 (0%) |
|  | ≤ -10 | 0 (0%) | 0 (0%) |
|  | ≤ -15 | 0 (0%) | 0 (0%) |

Baseline (time point = 0 Hour) is defined as the last measurement prior to Day 1 dosing. Change from baseline is the absolute difference between the measured value at each time point after drug administration and the baseline value.
